# When Appendicitis Masks a Malignancy: A Case of Appendiceal Goblet Cell Adenocarcinoma

**DOI:** 10.7759/cureus.89868

**Published:** 2025-08-12

**Authors:** Bethany M Gardner, Anna Haymov, Robert Jones

**Affiliations:** 1 General Surgery, Olean General Hospital, Olean, USA; 2 Neurology and Neurosurgery, Lake Erie College of Osteopathic Medicine, Elmira, USA

**Keywords:** atypical appendicitis, carcinoma of the appendix, gastrointestinal oncology, goblet cell adenocarcinoma, surgical case report

## Abstract

Goblet cell adenocarcinoma (GCA) of the appendix is a rare, mucus-producing tumor that exhibits both mucinous and neuroendocrine differentiation. It is most commonly diagnosed in patients presenting with acute appendicitis, and the definitive diagnosis is typically made after pathological examination. GCA is associated with a high risk of metastasis, with common sites including the right colon, ileum, lymph nodes, peritoneum, and omentum, and ovarian metastases are particularly noted in women with stage 4 disease. This case study focuses on a 55-year-old female patient who presented with right lower quadrant pain and was diagnosed with GCA following an appendectomy. The patient then underwent a complete right hemicolectomy as definitive therapy. This case highlights the challenges of diagnosing GCA, the importance of timely intervention, and the current treatment strategies.

## Introduction

Appendiceal tumors are uncommon, comprising less than 1% of all gastrointestinal malignancies, with goblet cell adenocarcinoma (GCA) representing an exceptionally rare subtype, accounting for fewer than 1% of all appendiceal cancers [1]. GCA is a distinctive neoplasm characterized by dual differentiation: mucin-producing glandular features alongside neuroendocrine elements [2-3]. This hybrid nature contributes to both its diagnostic challenges and its clinical behavior, which lies between conventional adenocarcinomas and neuroendocrine tumors in terms of aggressiveness and prognosis. Reported five-year survival rates range from 40% to 80%, reflecting this intermediate prognosis [1].

Most cases of GCA are discovered incidentally during procedures such as appendectomy, colonoscopy, or cross-sectional imaging [2]. When symptomatic, GCA typically mimics acute appendicitis, presenting with right lower quadrant abdominal pain, nausea, and vomiting. These signs are often attributed to inflammatory changes within the appendix; however, in cases of GCA, the neuroendocrine component may contribute to symptoms via serotonin-mediated mechanisms, particularly nausea and gastrointestinal dysmotility [3]. Gross intraoperative findings are often indistinguishable from simple appendicitis, which further obscures suspicion of malignancy. As a result, definitive diagnosis frequently relies on postoperative histopathologic evaluation [2].

This report presents a rare case of biopsy-confirmed GCA of the appendix in a patient who initially presented with classic symptoms of appendicitis. The case highlights the importance of considering GCA in the differential diagnosis, especially in older adults or when the clinical course or pathology deviates from typical expectations.

## Case presentation

A 55-year-old female patient with a medical history of chronic obstructive pulmonary disease, hypertension, chronic cough, alcohol abuse, and previous surgeries, including an open cholecystectomy and tubal ligation, presented to the emergency department with a three-day history of abdominal pain with migration to the right lower quadrant. She had associated nausea, vomiting, and constipation. On physical examination, she was found to be afebrile and had tenderness in the right lower quadrant but without rebound tenderness. Laboratory results revealed an elevated white blood cell (WBC) count of 13 K/mL with a left shift of 81.6% and an initial lactic acid level of 2.0 mmol/L (Table 1).

**Table 1:** Laboratory Evaluation of Patient Status Compared to Accepted Values

Test Name	Patient’s Value	Reference Range
White Blood Cell Count (K/mm³)	13	4-10.5
Red Blood Cell Count (M/µL)	4.16	4.2-5.4
Hemoglobin (gm/dL)	14.5	12.5-16
Hematocrit (%)	43	37-47
Platelet Count (K/mm³)	295	150-450
Neutrophil (%)	81.6	40-74
Lymphocyte (%)	9.8	19-48
Monocyte (%)	7.5	03-Sep
Eosinophil (%)	0.2	0-7
Basophil (%)	0.9	0-2
Glucose (mg/dL)	105	74-106
Blood Urea Nitrogen (mg/dL)	18	Sep-23
Creatinine (mg/dL)	1.21	0.55-1.02
Sodium (mmol/L)	138	136-145
Potassium (mmol/L)	3.7	3.5-5.1
Chloride (mmol/L)	106	98-110
Carbon Dioxide (mmol/L)	23.9	20-31
Anion Gap	8.1	Apr-13
Calcium (mmol/L)	9.9	8.7-10.4
Lactic Acid (mmol/L)	2	0.5-2.2

**Figure 1:**
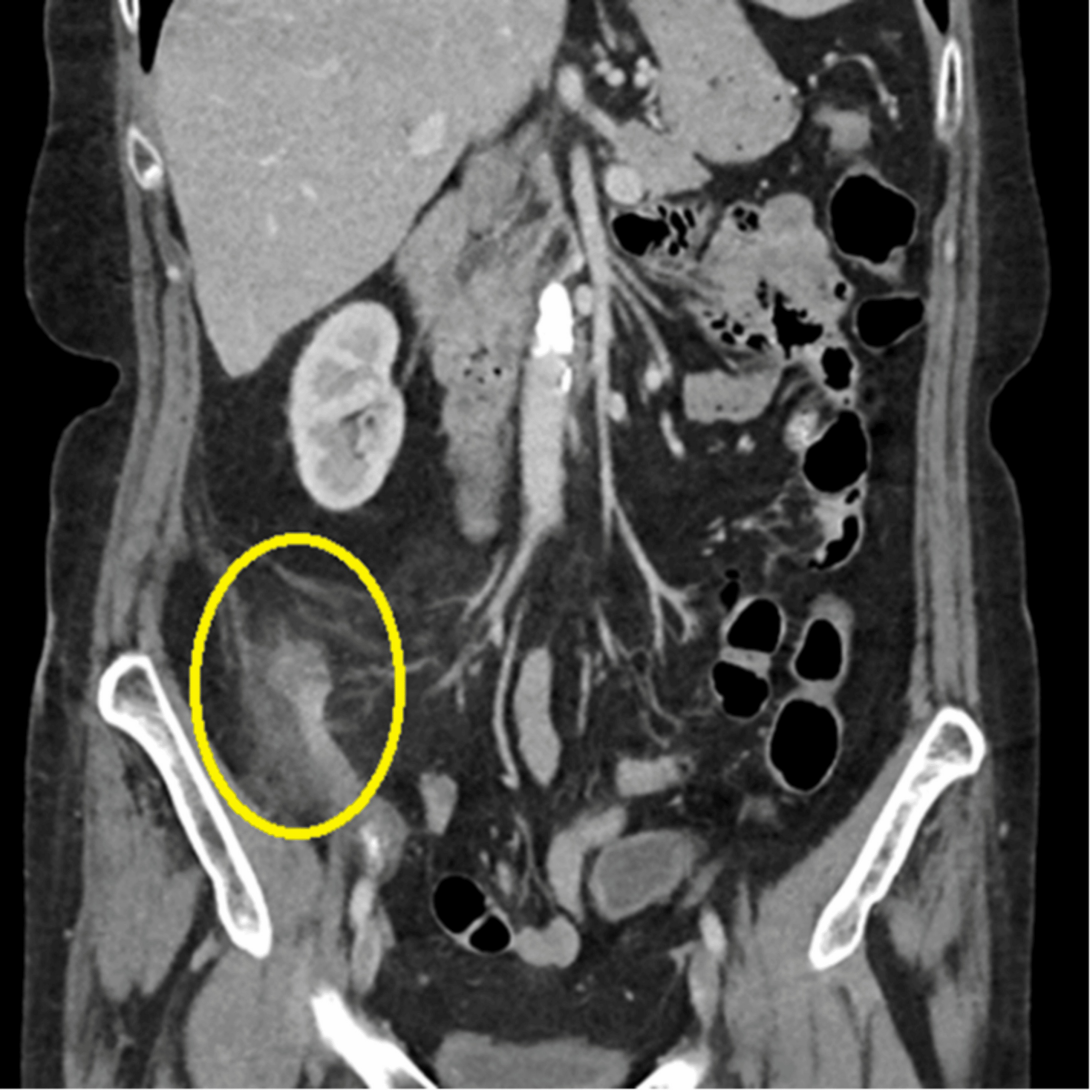
Coronal CT with contrast imaging of the abdomen Circled in yellow is the thickened appendix with extensive inflammatory changes surrounding the appendix, especially evident in the distal tip.

Her computed Appendicitis Inflammatory Response (AIR) score was six, which is indeterminate of appendicitis, and a definitive diagnosis depends on further imaging and observation. While less specific than AIR, her Alvarado score was eight, indicating a likely appendicitis. A CT scan further supported the diagnosis of appendicitis by showing thickening of the appendix, with significant inflammatory changes (Figure 1). 

**Figure 2:**
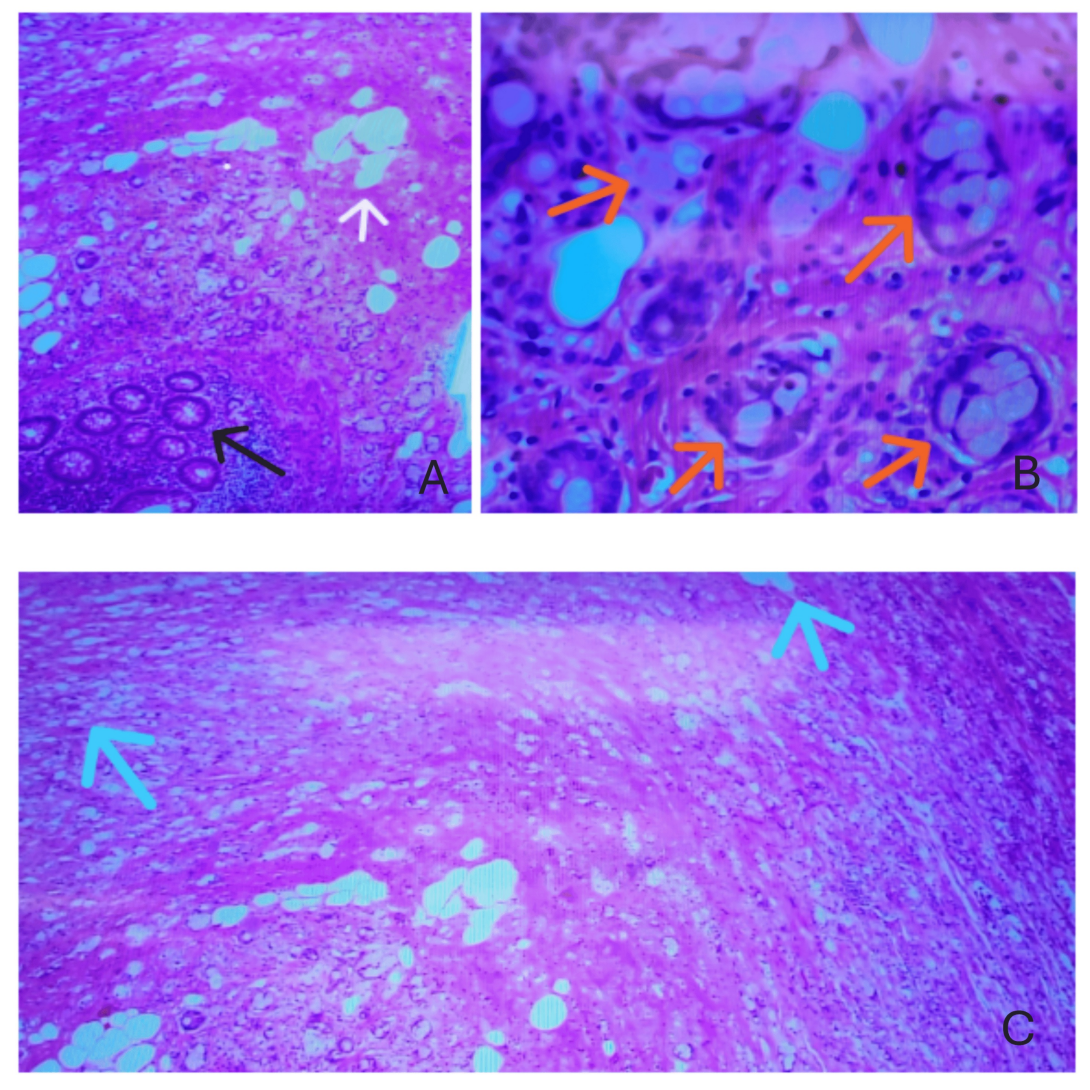
Histological slides taken from the appendix Image A: The black arrow indicates the presence of glands, and the white arrow shows goblet cells. Image B: The top right image shows signet ring goblet cells indicated with orange arrows; Image C: The light blue arrows show infiltration extending into the serosa.

After consultation with general surgery, the patient underwent a laparoscopic appendectomy. During surgery, the appendix appeared erythematous and edematous but intact with no evidence of rupture. It was relatively long and adhered to the cecum and abdominal wall. The mesoappendix appeared bulky and foreshortened. The pathology report measured the appendix to be 5 cm in length and 1.3 cm in diameter, with a wall thickness of 4 mm. The serosal surface and mucosa appeared gray-tan to red-brown, glistening, and slightly congested. The distal 2 cm of the appendix showed induration but no distinct mass. Microscopic analysis showed a mucinous tumor with signet ring cell features in the distal appendix, which invaded through the muscularis propria and extended to the serosal surface. Histological analysis of the specimen revealed nests of mucin-producing goblet cells infiltrating the submucosa and muscularis propria, with occasional solitary cells scattered throughout (Figure 2). Differentiation was observed based on the extent of nesting and trabecular structure, classifying the tumor as grade 2. The tumor morphology was most consistent with GCA, prompting the recommendation for further immunohistochemical evaluation. The proximal resection margin was clear. Additional findings included acute appendicitis with transmural inflammation and localized peritonitis. Images from the histological specimen are evident below (Figure 2). 

Immunohistochemical studies performed by the pathologist confirmed the diagnosis of GCA. Immunostains for CAM 5.2 were positive, highlighting the neoplastic cells, and SATB2, CDX2, and synaptophysin were also positive. The Ki-67 index was approximately 65%, indicating a high proliferative rate. Staging studies were performed, including a CT scan that was negative, and an MRI of the liver showed no acute disease, suggesting no evidence of metastatic disease.

A complete right hemicolectomy was performed a few weeks after the pathology report for further lymph node sampling and evaluation of potential local spread. Pathology reported no evidence of metastases in the resected colon or omentum. The 10 lymph nodes collected were negative for malignancy. This gave the tumor a pathologic stage of pT3N0M0 or stage IIA. At this stage, the right hemicolectomy was considered curative, and no additional treatment was planned. The patient will continue to follow up with the oncology clinic with occasional CT imaging. 

## Discussion

GCA of the appendix is a rare and distinct subtype of primary appendiceal cancer characterized by both mucinous and neuroendocrine differentiation [3]. This type of tumor accounts for less than 1% of appendiceal cancers but is notable for its potential for aggressive behavior and high risk of metastasis [1,4]. In fact, up to 50% of patients with GCA may develop metastatic disease, with common sites including the right colon, ileum, lymph nodes, peritoneum, and omentum [1,3]. In advanced stages, ovarian metastases are frequently observed, especially in women with stage 4 disease [1].

The diagnosis of GCA, particularly in early stages, is crucial, as its prognosis can vary significantly depending on the extent of the disease [1-2]. Patients with localized tumors typically have a favorable prognosis, with a five-year survival rate of approximately 86% when lymph node involvement is absent [2]. However, the prognosis deteriorates significantly in the presence of lymph node metastasis, with survival rates dropping to 42% in node-positive cases [2]. The involvement of lymph nodes is particularly associated with poorer long-term survival, highlighting the critical role of lymph node sampling in managing GCA [1-3].

GCA may also be graded according to histopathological findings. Complex anastomosing tubules, cribriform architecture, and confluent solid sheets characterize high-grade GCA [5]. It exhibits large irregular clusters, poorly cohesive goblet-like or nonmucinous cells, and a single-file growth pattern. Cytologically, it displays numerous mitoses, atypical mitotic figures, necrosis, and a desmoplastic stromal reaction, with adenocarcinomatous glands floating in mucin pools. Low-grade features include goblet-like mucinous cells arranged in a tubular pattern or small discrete clusters, with occasional endocrine and Paneth-like cells [5]. It shows mild nuclear atypia, a low mitotic rate, and limited tubular fusion or simple trabecular growth. Extracellular mucin is often present, sometimes in abundant amounts [5].

As this case demonstrates, a right hemicolectomy is considered the standard first-line treatment for GCA, even after the initial appendectomy [1-3]. Both North American and European neuroendocrine tumor societies recommend this approach due to the high risk of metastasis and the improvements in prognosis when this additional surgical step is taken [1,6]. Right hemicolectomy and mesenteric nodal resection are recommended for T3 or T4 disease, clinically positive mesenteric nodes, or evidence of direct cecal extension [2]. For tumors that are localized, smaller than 2 cm, and have negative margins, an appendectomy alone may be sufficient, though this remains controversial [1,4].

Adjuvant chemotherapy is indicated for patients with stage III and stage IV disease, as well as those with recurrent disease [1,3]. Given the histological resemblance of metastatic GCA to colorectal adenocarcinoma, chemotherapy regimens commonly used for colorectal cancer, such as FOLFOX (5-FU, leucovorin, oxaliplatin) and FOLFIRI (5-FU, folic acid, irinotecan), are typically employed [1]. However, due to the rarity of GCA, large-scale randomized controlled trials are not feasible, and treatment guidelines are primarily based on anecdotal reports and small case series [3].

For patients with locally advanced or recurrent peritoneal disease, cytoreductive surgery with hyperthermic intraperitoneal chemotherapy (CRS + HIPEC) has been shown to improve median survival [1,6]. However, traditional therapies for metastatic carcinoids, such as interferon, somatostatin analogs, and targeted therapies like everolimus and sunitinib, are not effective for GCA due to the lack of adequate uptake on imaging studies such as Octreoscan or gallium-68 PET scans [1]. 

In this case study, immunohistochemical findings were positive for CAM 5.2, SATB2, CDX2, and synaptophysin [1]. The elevated Ki-67 index suggests an increased proliferative rate (1). The prognostic value of Ki-67 in GCA remains a subject of ongoing research, as some studies suggest worsening survival rates with increasing Ki-67 [7], although the significance remains unclear [1]. Lifelong surveillance is recommended; however, the frequency and additional testing depend on the staging of the tumor and which measures were taken for removal [1]. 

## Conclusions

GCA of the appendix is a rare but aggressive malignancy that often presents with nonspecific symptoms, making early diagnosis challenging. In this case, the patient’s diagnosis was confirmed after an appendectomy, and further staging revealed no metastasis or lymph node involvement, allowing for successful treatment with right hemicolectomy alone. Although the prognosis for GCA can be favorable when diagnosed at an early stage, the presence of metastases significantly worsens the outlook, underscoring the importance of early and thorough staging. Treatment typically involves aggressive surgical management, including right hemicolectomy, particularly for tumors with higher risk features. Adjuvant chemotherapy is indicated for advanced or recurrent disease, and ongoing research is necessary to refine treatment protocols. This case highlights the critical need for a high level of clinical suspicion and the role of early intervention in improving survival outcomes for patients with GCA of the appendix.
